# NMR-based metabonomic analysis of HUVEC cells during replicative senescence

**DOI:** 10.18632/aging.102834

**Published:** 2020-02-17

**Authors:** Shenghui Yi, Kejiang Lin, Ting Jiang, Wei Shao, Caihua Huang, Bin Jiang, Qinxi Li, Donghai Lin

**Affiliations:** 1 College of Chemistry and Chemical Engineering, Key Laboratory for Chemical Biology of Fujian Province, MOE Key Laboratory of Spectrochemical Analysis and Instrumentation, Xiamen University, Xiamen 361005, China; 2 Department of Medical Chemistry, China Pharmaceutical University, Nanjing 210009, China; 3 Research and Communication Center of Exercise and Health, Xiamen University of Technology, Xiamen 361024, China; 4 State Key Laboratory of Cellular Stress Biology, School of Life Science, Xiamen University, Xiamen 361102, China

**Keywords:** HUVECs, replicative senescence, metabolomics, NMR spectroscopy

## Abstract

Cellular senescence is a physiological process reacting to stimuli, in which cells enter a state of irreversible growth arrest in response to adverse consequences associated with metabolic disorders. Molecular mechanisms underlying the progression of cellular senescence remain unclear. Here, we established a replicative senescence model of human umbilical vein endothelial cells (HUVEC) from passage 3 (P3) to 18 (P18), and performed biochemical characterizations and NMR-based metabolomic analyses. The cellular senescence degree advanced as the cells were sequentially passaged *in vitro*, and cellular metabolic profiles were gradually altered. Totally, 8, 16, 21 and 19 significant metabolites were primarily changed in the P6, P10, P14 and P18 cells compared with the P3 cells, respectively. These metabolites were mainly involved in 14 significantly altered metabolic pathways. Furthermore, we observed taurine retarded oxidative damage resulting from senescence. In the case of energy deficiency, HUVECs metabolized neutral amino acids to replenish energy, thus increased glutamine, aspartate and asparagine at the early stages of cellular senescence but decreased them at the later stages. Our results indicate that cellular replicative senescence is closely associated with promoted oxidative stress, impaired energy metabolism and blocked protein synthesis. This work may provide mechanistic understanding of the progression of cellular senescence.

## INTRODUCTION

Aging is characterized by a progressive loss of physiological integrity, leading to impaired functions and increased vulnerability to death [1]. Numerous lines of evidence indicate that the senescence of endothelial cells is an initiating factor of human aging, which plays a pivotal role in the development of age-related diseases such as cardiovascular disorders and diabetes. During senescence, endothelial cells undergo a series of changes at cellular and molecular levels. The senescent endothelial cells appear enlarged and flattened. Apart from the morphological changes, they also possess abnormal physiological functions, encompassing the loss of proliferative capacity, apoptosis, G1 phase arrest, morphological and functional alterations of the mitochondria, etc. [2, 3]. Additionally, senescent cells also exhibit increased levels of oxidatively modified proteins, elevated activity of senescence-associated β-galactosidase, and shortened length of the telomere DNA at the molecular level. Moreover, the 50 RNAs are always elevated and 18 RNAs are consistently reduced in senescent cells, including many protein-coding mRNAs and some non-coding RNAs [4]. Although senescence-related changes are extremely complicated, they are well reflected by the alterations in transcriptome [5, 6], proteome [7] and ultimately metabolome [8]. Previous works have identified several cellular and molecular hallmarks of aging, including: genomic instability, telomere attrition, epigenetic alterations, loss of proteostasis, deregulated nutrient sensing, mitochondrial dysfunction, cellular senescence, stem cell exhaustion, and altered intercellular communication [1]. Each of the nine hallmarks is closely connected to undesirable metabolic changes. Furthermore, it is indicated that several metabolic alterations accumulate over time along with a reduction in biological fitness, suggesting the existence of a “metabolic clock” that controls aging [9]. Expectedly, metabolisms and metabolic control plays crucial roles in aging [9].

As downstream reflections of transcription and translation alterations, metabolic changes significantly contribute to the molecular mechanisms of aging [8]. Metabolomic analyses have been previously performed to address aging-related metabolic changes [10]. For example, Lawton et al. reported 51 metabolites greatly correlated with human aging [11], Yu, Z. et al. found that human serum metabolic profiles were age-dependent [12], and Menni et al. identified metabonomic makers to map new aging-related metabolic pathways in human populations [13]. However, molecular mechanisms underlying progressive aging remain elusive. As aging is usually associated with distinct manners and rates for different individuals [8], the aging-related heterogeneity on human beings increases the difficulties to elucidate the common molecular mechanisms from cohort study with metabolomic analyses. On the other hand, *in vitro* cell aging models have been widely used in aging-related studies [14]. Several previous studies have reported significant metabolic changes related to cell aging. Zwerschke et al. showed that carbohydrate metabolism was drastically suppressed in senescent cells, characterized by both impaired glycolytic enzyme activities and abnormal ATP levels [15]. Unterluggauer et al. found that glutamine was greatly consumed in senescent human umbilical vein endothelial cells (HUVECs), indicating the important role of glutaminolysis in cellular senescence [16]. So far, few comprehensive metabolomic analyses have been conducted to mechanistically understand metabolic changes during the progression of cellular senescence.

Recently, two cellular senescence models have been used to study aging-related issues *in vitro*, including stress-induced premature senescence (SIPS) and replicative senescence (RS). The RS phase represents a state of indefinite growth arrest of cells after a certain number of cumulative population doublings that was first described by Hayflick [17]. RS can be induced by various sublethal stresses, including telomere shortening, genomic injury, epigenomic damage and signaling from oncoproteins [4]. Differently, the SIPS phrase describes the long-term response of cells to long-term subcytotoxic stress. A previous work indicated that the long-term subcytotoxic stress could also induce RS, but SIPS and RS are substantially distinct phenotypes with different expressions of proteins even though both share some of phenotypic features [18]. In view of the fact that aging is accompanied by a time-dependent alteration during cell doubling, RS is much closer to natural cell aging process than SIPS [18]. In the present work, we established a replicative senescence model by serial passaging of the primary HUVEC cells, and performed metabolomic analysis to explore cellular metabolic profiles as the cells progressed towards senescence. We identified significant metabolites primarily responsible for metabolic discrepancies among the HUVEC cells at different passages, and mapped significantly altered metabolic pathways during the progression of cellular replicative senescence. This work sheds light on the molecular mechanisms of cellular replicative senescence.

## RESULTS

### Biochemical characteristics of HUVECs during replicative senescence

**Figure 1:**
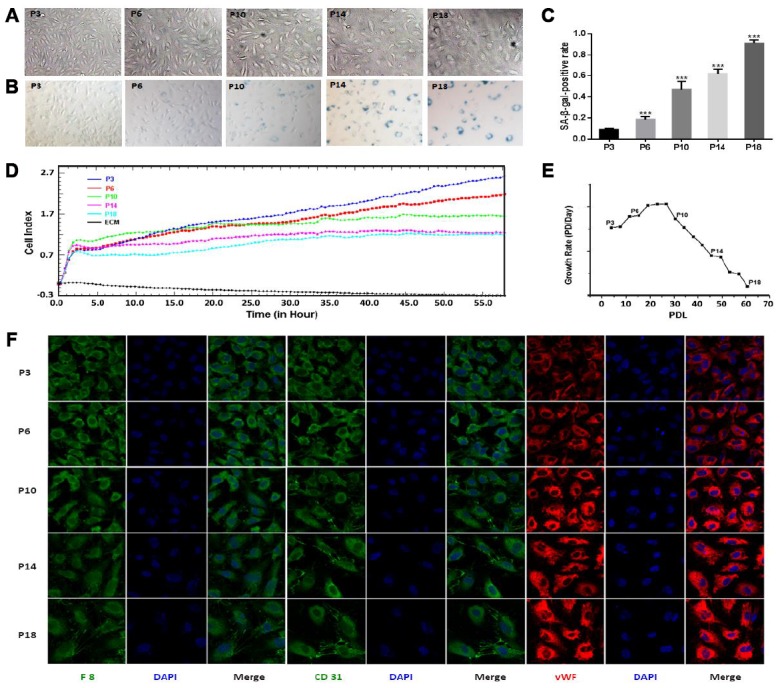
**Characterization of senescence in HUVEC cells during continual passaging.** (**A**) Cell morphological characteristics (×100 magnification). (**B**) Senescence-associated-galactosidase (SA-β-gal) staining (×100 magnification). (**C**) Percentages of SA-β-gal-positive cells. Data were presented by means ± SE, *** denotes the statistical significance *p* < 0.001 relative to the P3 group. (**D**) Real-time cell growth curves. The initial 4 or 5 h was the time for cell adherence. (**E**) Cell growth rates. (**F**) Cell immunofluorescence assay (×400 magnification). Positive immunoreactivity is shown for several endothelial markers including anti-F8 (green), CD31 (green) and von Willebrand factor (vWF; red) antibodies. Nuclei were stained with 4’, 6-diamidino-2-phenylindole (DAPI) as a contrast (blue).

**Figure 2:**
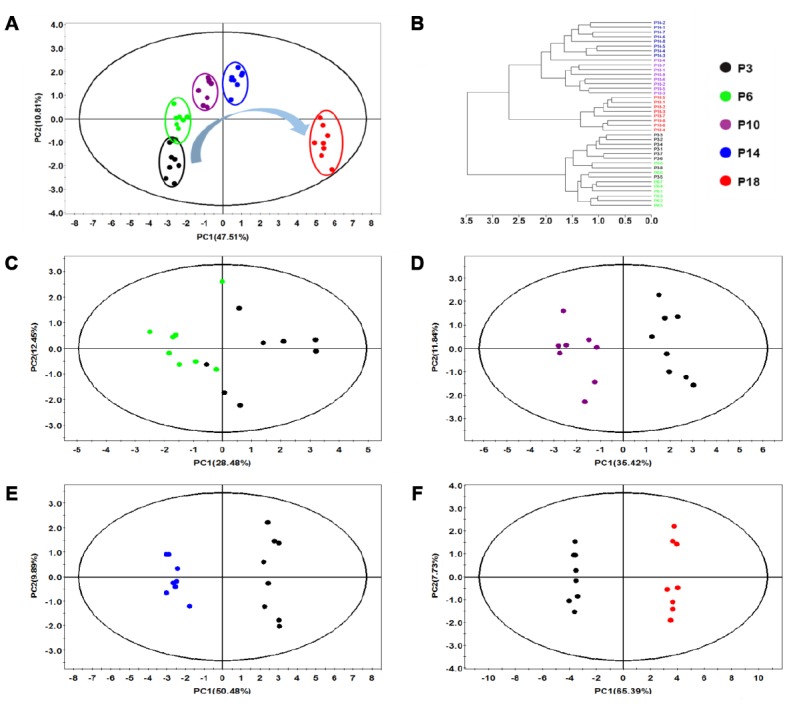
**Multivariate analysis of changed metabolic profiles of HUVEC cells during continued passaging.** (**A**) PCA scores plot of ^1^H NMR data obtained from the five groups of cells. (**B**) Hierarchical cluster analysis of the five groups of cells. (**C**–**F**) Pair-wise PCA scores plots of P6 cells vs. P3 cells (**C**), P10 cells vs. P3 cells (**D**), P14 cells vs. P3 cells (**E**), P18 cells vs. P3 cells (**F**).

**Figure 3:**
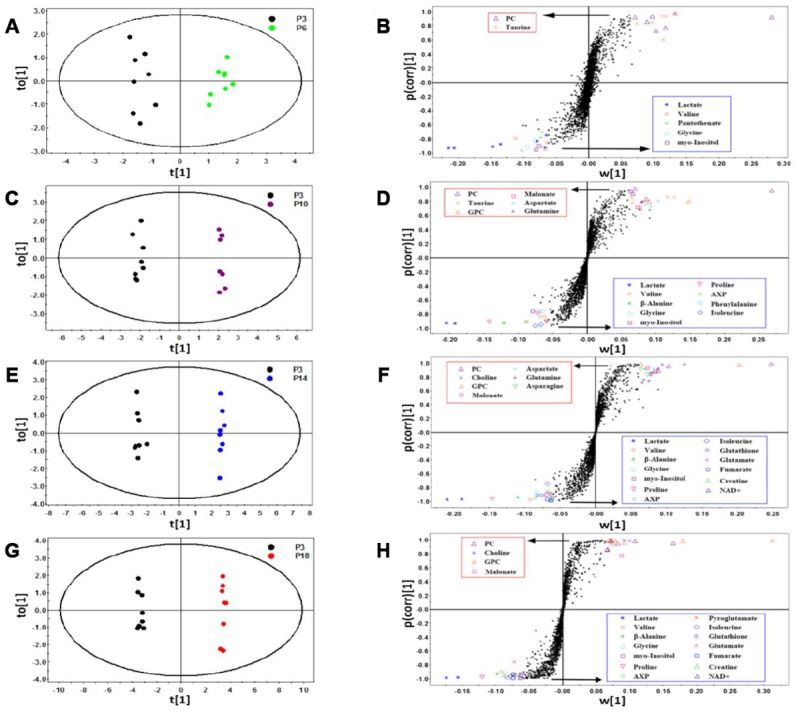
**Identification of significant metabolites primarily responsible for distinguishing metabolic profiles of the four groups of HUVEC cells from the P3 group.** (**A**–**H**) OPLS-DA scores plots and corresponding S-plots of P6 vs. P3 (**A** and **B**), P10 vs. P3 (**C** and **D**), P14 vs. P3 (**E** and **F**), P18 vs. P3 (**G** and **H**). Each point in the OPLS-DA scores plots represents a cell sample. Each dot in the S-plots denotes a bin. Bin points with |w [1] | > 0.06, |p(corr) [1] | > 0.75 and VIP ≥ 1.00, were identified to be significant metabolites. The identified metabolites showed in the red/blue rectangle represent significantly up-regulated/down-regulated metabolites in the four groups of cells relative to the P3 group.

To establish the cellular replicative senescence model, HUVEC cells underwent a total of 61 PDL till passage 18 (P18). The PDL values were 4, 15, 31 and 46 for passages 3, 6, 10, 14 (P3, P6, P10 and P14), respectively. Cellular senescence was evaluated by a combination of morphology observation, SA-β-gal assay and proliferation detection. Consistently with previous studies [19], HUVECs at early passages were characterized as adherent cells exhibiting a spindle and round shaped cobblestone appearance, and were progressively changed to a large and flattened shape exhibiting sparse arrangement at later passages (Figure 1A). The replicative senescence of HUVECs was further confirmed by the following results obtained from SA-β-gal assay and proliferation detection: 1) HUVECs exhibited increasing positive rates of SA-β-gal-staining cells from 9% up to 91% during serial subcultivation (Figure 1B, 1C); 2) the CI values of the cells at the later passages (P10, P14 and P18) were lower than those at the early passages (P3, P6) (Figure 1D); 3) HUVECs showed declining growth rates as the cells were continually passaged (Figure 1E). Taken together, HUVECs exhibited a progressive change of morphology, a gradual decrease in proliferative capacity and a successive rise of SA-β-galactosidase activity during the prolonged *in vitro* cultivation. These observations were indicative of the replicative senescence of HUVECs.

Given that endothelial-mesenchymal transition (EndMT) is an adaptive response of endothelial cells to either chronic stress or the change of microenvironment, the possibility of the occurrence of EndMT had to be eliminated during cellular replicative senescence. We thus examined the endothelial phenotype of HUVECs by analyzing expressions of several typical makers in HUVECs (F8, CD31 and vWF). The positive expressions of the three markers indicated that EndMT did not occur in our experiments (Figure 1F), implying that the replicative senescence model used here was valid. During the establishment and evaluation of the cellular replicative senescence model, we observed an interesting phenomenon that P10 appeared to be a turning point during the progression of HUVECs senescence. Significantly, HUVECs at P3 and P6 showed similar morphologies, while those at P10 displayed a distinctly different morphology (Figure 1A, 1B). Furthermore, nearly half of HUVECs at P10 were positive for SA-β-gal staining (Figure 1C), whereas HUVECs at P3 and P6 were low positive percentages. In addition, the slope of cell growth curve of P10 was explicitly lower compared to those of P3 and P6, but similar to those of P14 and P18 (Figure 1D).

### Metabolic profiles of HUVECs during replicative senescence

The typical 1D ^1^H NMR spectra of aqueous extracts derived from HUVEC cells at different passages are displayed in Supplementary Figure 1. The typical 2D ^1^H-^13^C HSQC and ^1^H-^1^H TOCSY spectra of aqueous extracts derived from the P3 cells are shown in Supplementary Figures 2–5, respectively, which were used to confirm the resonance assignments of metabolites. Totally, 33 metabolites were unambiguously identified based on the NMR spectra, allowing us to address metabolic changes in the HUVEC cells at different passages.

At first, the PCA analysis of the five groups of cells was performed to obtain a comprehensive comparison of metabolic profiles in HUVECs during replicative senescence. The resultant PCA scores plot shows distinct metabolic separations among the five groups (P3, P6, P10, P14 and P18) and a changing trend in metabolic profiles from P3 to P18 (Figure 2A). Furthermore, this plot exhibits a well-clustered pattern for the five groups of cells. All HUVEC cells were roughly classified into three clusters. The P3 and P6 groups were somewhat partially overlapped, the P10 and P14 groups were closer to each other, and the P18 group was clearly separated from the other four groups. Furthermore, Hierarchical cluster analysis supported the result from the PCA analysis. As shown in Figure 2B, the P3 and P6 cells were grouped into the same cluster, the P10 and P14 cells belonged to another cluster, and the P18 cells formed a separate cluster.

Then, pairwise PCA analyses were performed to assess the changes of metabolic profiles in HUVEC cells during the continuous passage relative to the P3 cells. The results exhibited that the P6 cells was metabolically discriminated from the P3 cells (Figure 2C), and the P10, P14 and P18 cells were distinctly different from the P3 cells (Figure 2D–2F). It seemed that the continuously changing metabolic profiles of the cells were closely related to the progressive phenotypes of senescent cells. The more times the cells were passaged, the farther the metabolic profiles of the cells were from that of the P3 cells (Figure 2). The aging-dependent metabolic changes were further characterized by the explained variances of the first principal component (PC1), which were calculated from the pairwise PCA models: P6 vs. P3, 28.48%; P10 vs. P3, 35.42%; P14 vs. P3, 50.48%; P18 vs. P3, 65.39% (Figure 2C–2F).

In summary, the metabolomic analysis demonstrated that the metabolic profiles of HUVECs were continuously changed during the progression of cellular replicative senescence, supporting the biochemical characterization of replicative senescence of HUVECs described above. The P10, P14 and P18 groups displayed similar metabolic profiles, but distinctly different metabolic profiles from the P3 and P6 groups, which suggested that P10 might act as a critical turning point in the changing metabolic process of cellular replicative senescence.

### Significant metabolites in HUVECs during replicative senescence

We conducted supervised OPLS-DA analyses to explore the variables significantly responsible for discriminating the metabolic profiles of the four groups (P6, P10, P14 and P18) from that of the P3 group. The OPLS-DA score plots exhibit that the four groups were clearly discriminated from the P3 group (Figure 3A, 3C, 3E, 3G). Supplementary Table 1 shows the corresponding parameters used to assess the qualities of the OPLS-DA models. The higher the R^2^Y and Q^2^ values were, the more credible and robust the OPLS-DA model was.

Based on the S-plots of the OPLS-DA models (Figure 3B, 3D, 3F, 3H), we identified significant metabolites primarily responsible for the separation of metabolic profiles between the P3 group and the other four groups. These significant metabolites are shown in Supplementary Tables 2. Totally, 7, 15, 20 and 18 significant metabolites were identified for P6 vs. P3 (Figure 3B, 2 increased and 5 decreased metabolites), P10 vs. P3 (Figure 3D, 6 increased and 9 decreased metabolites), P14 vs. P3 (Figure 3F, 7 increased and 13 decreased metabolites), and P18 vs. P3 (Figure 3H, 4 increased and 14 decreased metabolites), respectively.

Moreover, we calculated relative integrals of the metabolites, and performed one-way ANOVA analyses to quantitatively compare the metabolite levels among the five groups of HUVECs. The metabolites with statistical significance *p* < 0.05 were identified to be differential metabolites (Table 1). The differential metabolites identified from the univariate analyses were almost consistent with the significant metabolites identified from the S-plots of the OPLS-DA models (Figure 3, Supplementary Table 2). In detail, 7, 14, 21, 19 differential metabolites were identified in the P6, P10, P14 and P18 groups relative to the P3 group, respectively. The 7 differential metabolites in the P6 group were fully identical to the above-described significant metabolites. Compared with the P3 group, the P10 group had one extra significant metabolite (glutamine with a low VIP of 1.009), the P14 group had one extra differential metabolite (taurine with a higher *p* < 0.005), and the P18 group had also one extra differential metabolite (aspartate with a higher *p* < 0.005).

**Table 1:** One-way ANOVA for comparing the levels of differential metabolites among the five groups of HUVEC cells.

**Metabolites**	**Tukey's multiple comparison test**								**Mean ± Std. Error**						**One-way ANOVA**	
	**P6**	**P10**	**P14**	**P18**	**P10**	**P14**	**P18**		**P3**	**P6**	**P10**	**P14**	**P18**		**F**	***p***
	**vs.**	**vs.**	**vs.**	**vs.**	**vs.**	**vs.**	**vs.**									
	**P3**	**P3**	**P3**	**P3**	**P6**	**P10**	**P14**									
Pantothenate	***	ns	ns	ns	ns	ns	*		0.172 ±	0.155 ±	0.163 ±	0.163 ±	0.178 ±		12.836	1.56e-06
									0.002	0.002	0.002	0.003	0.003			
Isoleucine	ns	*	**	***	ns	ns	ns		0.250 ±	0.235 ±	0.222 ±	0.216 ±	0.195 ±		13.443	9.79e-07
									0.005	0.005	0.006	0.004	0.008			
Valine	*	***	***	***	ns	ns	ns		0.579 ±	0.538 ±	0.510 ±	0.500 ±	0.477 ±		19.584	1.54e-08
									0.006	0.009	0.009	0.006	0.013			
Lactate	**	***	***	***	*	**	***		3.215 ±	2.352 ±	1.794 ±	1.260 ±	0.890 ±		100.720	1.07e-18
									0.131	0.108	0.098	0.032	0.049			
Proline	ns	***	***	***	***	ns	***		0.683 ±	0.670 ±	0.544 ±	0.505 ±	0.375 ±		105.962	4.75e-19
									0.013	0.010	0.018	0.011	0.008			
Glutathione	ns	ns	***	***	ns	ns	***		1.273 ±	1.234 ±	1.169 ±	1.056 ±	0.774 ±		63.801	1.38e-15
									0.030	0.014	0.034	0.026	0.015			
Pyroglutamate	ns	ns	ns	**	*	ns	ns		1.297 ±	1.346 ±	1.126 ±	1.118 ±	0.993 ±		12.287	2.42e-06
									0.051	0.058	0.027	0.035	0.025			
Glutamate	ns	ns	***	***	**	***	***		8.501 ±	8.643 ±	8.278 ±	7.741 ±	6.670 ±		153.993	1.07e-21
									0.091	0.064	0.036	0.055	0.064			
Glutamine	ns	ns	***	ns	***	***	***		4.854 ±	4.855 ±	5.167 ±	5.674 ±	4.857 ±		36.949	4.14e-12
									0.089	0.033	0.035	0.021	0.082			
Aspartate	ns	***	***	*	**	ns	***		0.784 ±	0.818 ±	0.873 ±	0.862 ±	0.721 ±		43.126	4.62e-13
									0.013	0.008	0.009	0.005	0.009			
Asparagine	ns	ns	***	ns	ns	ns	***		0.221 ±	0.225 ±	0.238 ±	0.260 ±	0.221 ±		14.571	4.23e-07
									0.003	0.002	0.007	0.001	0.005			
Creatine	ns	ns	***	***	*	***	***		0.894 ±	0.902 ±	0.878 ±	0.789 ±	0.691 ±		326.782	3.48e-27
									0.004	0.004	0.006	0.005	0.006			
Malonate	ns	*	***	***	ns	ns	**		0.574 ±	0.576 ±	0.642 ±	0.700 ±	0.823 ±		38.965	1.96e-12
									0.012	0.017	0.016	0.017	0.020			
β-Alanine	ns	***	***	***	***	***	ns		0.210 ±	0.202 ±	0.161 ±	0.117 ±	0.109 ±		73.273	1.63e-16
									0.003	0.004	0.006	0.004	0.009			
Choline	ns	ns	***	***	ns	***	***		0.309 ±	0.312 ±	0.333 ±	0.469 ±	0.651 ±		226.682	1.69e-24
									0.008	0.008	0.010	0.009	0.014			
PC	***	***	***	***	***	***	ns		3.150 ±	3.643 ±	4.076 ±	4.453 ±	4.626 ±		134.370	1.00e-20
									0.034	0.040	0.037	0.026	0.093			
GPC	ns	**	***	***	***	***	***		2.189 ±	2.183 ±	2.582 ±	3.206 ±	6.290 ±		1246.712	3.31e-37
									0.041	0.034	0.063	0.024	0.067			
Taurine	**	***	*	ns	ns	ns	*		2.115 ±	2.239 ±	2.325 ±	2.214 ±	2.102 ±		16.143	1.40e-07
									0.020	0.020	0.033	0.019	0.020			
myo-Inositol	*	***	***	***	*	ns	ns		0.109 ±	0.089 ±	0.067 ±	0.054 ±	0.048 ±		37.243	3.71e-12
									0.003	0.002	0.006	0.004	0.004			
Glycine	**	***	***	***	**	ns	***		0.748 ±	0.705 ±	0.653 ±	0.681 ±	0.623 ±		45.415	2.19e-13
									0.007	0.006	0.009	0.006	0.007			
Fumarate	ns	ns	***	***	ns	***	***		0.035 ±	0.035 ±	0.038 ±	0.027 ±	0.018 ±		99.676	1.27e-18
									0.001	0.001	0.001	0.001	0.001			
Phenylalanine	ns	*	ns	ns	ns	ns	ns		0.218 ±	0.204 ±	0.199 ±	0.208 ±	0.202 ±		2.676	0.048
									0.003	0.004	0.004	0.005	0.006			
AXP	ns	**	***	***	*	ns	ns		0.678 ±	0.666 ±	0.570 ±	0.567 ±	0.547 ±		17.465	5.79e-08
									0.012	0.015	0.018	0.014	0.014			
NAD^+^	ns	ns	***	***	ns	***	ns		0.120 ±	0.121 ±	0.116 ±	0.104 ±	0.099 ±		52.424	2.66e-14
									0.001	0.002	0.001	0.001	0.002			

Note: The Bonferroni-correction was conducted for the multiple comparison test. Symbols ***, **, *, ns mean differences are highly significant (*p* < 0.0001), very significant (*p* < 0.001), significant (*p* < 0.005), insignificant (*p* > 0.005), respectively. The metabolites with statistical significance *p* < 0.005 were identified to be differential metabolites. n=8 for each group. Red and blue colors denote that the difference is positive (i.e. increased metabolite in A relative to B) and negative, respectively.

**Figure 4:**
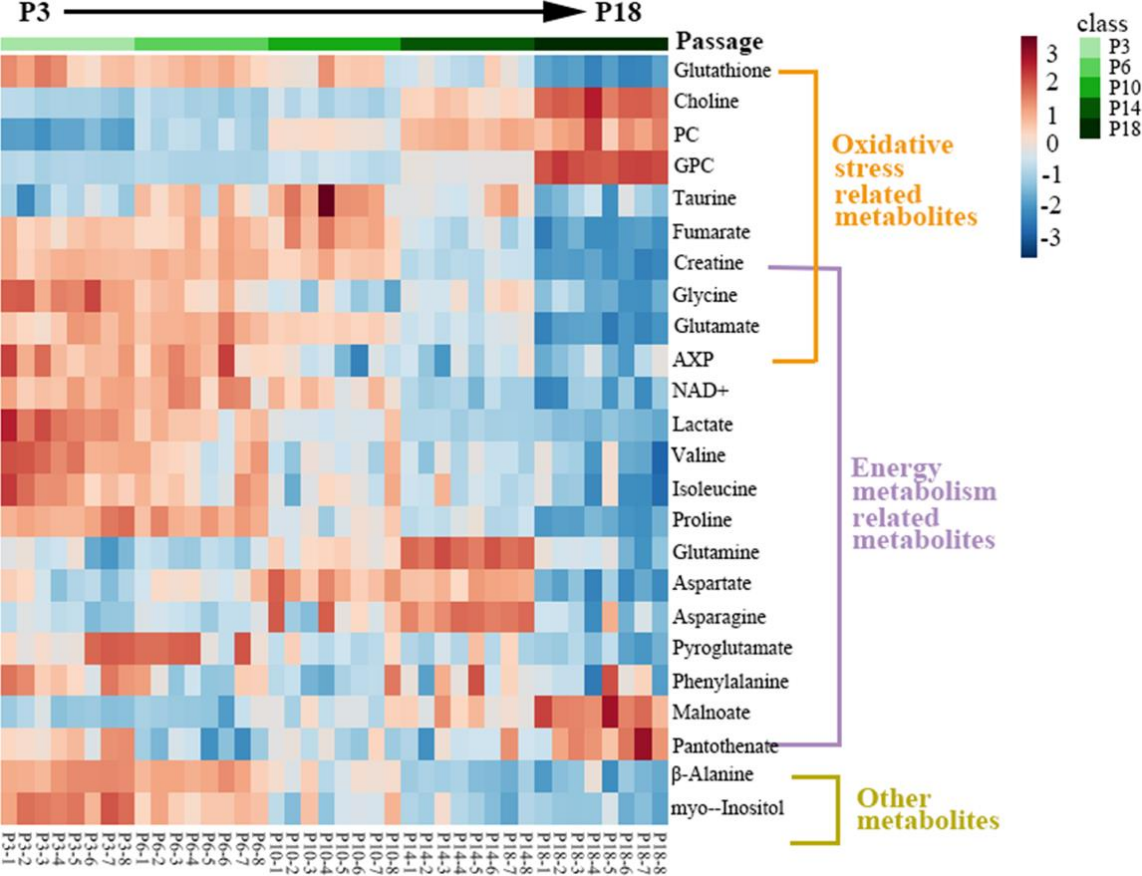
**Heatmap of significant metabolites in the five groups of HUVEC cells.** The significant metabolites were identified from the OPLS-DA S-plots of the four groups of cells vs. the P3 group. Red/blue colors indicate increased/decreased metabolite levels.

Furthermore, the relative levels of significant metabolites are displayed in a clustering heatmap, which shows that all metabolite levels were significantly changed as cells were continually passaged (Figure 4). Totally, 14 metabolite levels showed decreasing tendencies with the increase of cell passage, including isoleucine, valine, glutathione, fumarate, glutamate, creatine, glycine, AXP, pyroglutamate, β-alanine, NAD^+^, proline, lactate and myo-inositol. In contrast, 4 metabolite levels showed increasing tendencies, including PC, malonate, choline and GPC. Interestingly, several metabolite levels (taurine, aspartate, glutamine and asparagine) exhibited similar decreasing tendencies at earlier passages followed by increasing tendencies at latter passages, while the pantothenate level showed the contrary changing tendency during cellular replicative senescence.

Mostly, these changing metabolites were involved in metabolic pathways related to oxidative stress and energy metabolism. This result suggested that both the impaired redox equilibrium oxidative stress and disturbed energy metabolism significantly contributed to the replicative senescence of HUVECs.

### Significant metabolic pathways during replicative senescence

**Figure 5:**
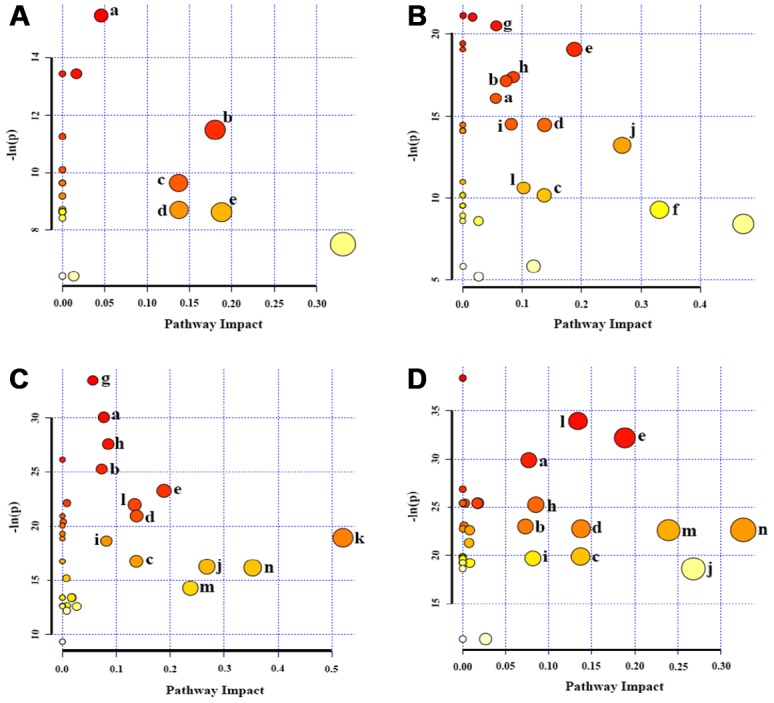
**Significantly altered metabolic pathways in the four groups of HUVEC cells compared to the P3 group.** (**A**) P6 vs. P3; (**B**) P10 vs. P3; (**C**) P14 vs. P3; (**D**) P18 vs. P3. A bubble represents an identified metabolic pathway. The bubble size is proportional to the pathway impact value (PIV), with the color denoting the statistical significance [-ln(*p*)] from highest (in red) to lowest (in white). Metabolic pathways with -ln(*p*) > 8 and PIV > 0.03 were identified to be significantly altered metabolic pathways, including: a, glycerophospholipid metabolism; b, pantothenate and CoA biosynthesis; c, inositol phosphate metabolism; d, pyruvate metabolism; e, glycine, serine and threonine metabolism; f, taurine and hypotaurine metabolism; g, aminoacyl-tRNA biosynthesis; h, propanoate metabolism; I, purine metabolism; j, β-Alanine metabolism; k, glanine, aspartate and glutamate metabolism; l, arginine and proline metabolism; m, glutathione metabolism; n, glutamate and glutamine metabolism.

**Figure 6:**
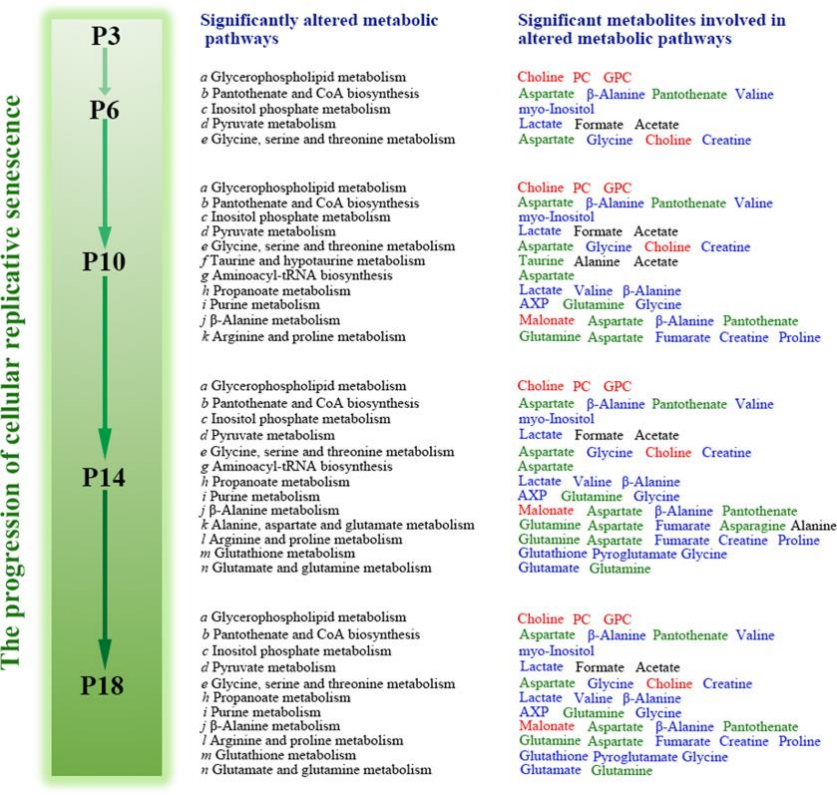
**Schematic representation of significantly altered metabolic pathways associated with the four groups of HUVEC cells compared with the P3 group.** Red, blue and black colors represent significantly increased, decreased and substantially unchanged metabolites during the progression of cellular replicative senescence relative to the early P3 passage. Green colors denote the metabolites with increasing tendencies followed by decreasing tendencies, or those with the contrary changing trends.

To identify significantly altered metabolic pathways related to the replicative senescence of HUVECs, we performed metabolic pathway analyses by using the MetaboAnalyst 4.0 webserver. We compared the four groups (P6, P10, P14 and P18) with the P3 group, and identified 5, 11, 13 and 11 significant metabolic pathways in the P6, P10, P14 and P18 groups, respectively (Figure 5). The five groups shared 5 significant metabolic pathways in the process of cellular replicative senescence, including: (a) glycerophospholipid metabolism; (b) pantothenate and CoA biosynthesis; (c) inositol phosphate metabolism; (d) pyruvate metabolism and glycine; (e) serine and threonine metabolism (Figure 6). Furthermore, the importances of these significant pathways were gradually increased during cellular senescence, as indicated by the rising -ln(*p*) scores (Figure 5). Besides the 5 pathways, several extra significant pathways were identified from metabolic comparisons of P10 vs. P3, P14 vs. P3 and P18 vs. P3, including: (f) taurine and hypotaurine metabolism; (g) aminoacyl-tRNA biosynthesis; (h) propanoate metabolism; (i) purine metabolism; (j) β-alanine metabolism; (l) arginine and proline metabolism; (m) glutathione metabolism; (n) glutamate and glutamine metabolism. Figure 6 displays the schematic overview of the significant metabolic pathways in HUVECs during replicative senescence together with the relevant metabolites involved in these pathways.

The number of the significant pathways identified in the P6 cells was smaller than those in the P10, P14 and P18 cells (11, 13, 11 vs. 5), implying the relatively smaller metabolic change in the P6 cells compared to the P3 cells. Compared with the P6 cells, the P10 cells showed 6 extra significant pathways (f-k). The cells at the latter passages during replicative senescence (P10, P14 and P18) showed almost identical numbers of significant pathways and shared 3 extra significant pathways (h, i, j) besides the above-described 5 significant pathways shared by all the five groups (a-e). Note that the P14 and P18 cells shared 6 extra significant pathways (h, i, j, l, m, n) except for the two significant pathways increased uniquely in the P14 cells (g, k). These results supported the multivariate pattern recognition analyses described above. As expected, HUVEC cells at the earlier passages (P3, P6) during replicative senescence were young and vigorous, while those in the latter passages (P10, P14 and P18) grew old and gradually lost their vitalities.

## DISCUSSION

Vascular aging, primarily characterized by dysfunction of endothelial cells, is a major risk factor for cardiovascular disease [20] and other aging-related diseases, such as degenerative diseases and metabolic syndrome. Cellular senescence is a complex and progressive event involving multiple processes and mechanisms. Even though a large number of evidence have proven the pivotal roles of dysfunction of endothelial cells, relatively little is known about metabolic changes in the process of vascular aging. Previous works have explored changes in energy metabolism in ageing cells [15, 16], and revealed the significances of metabolic control of longivity [9]. However, dynamic and comprehensive metabolic changes during the progression of cellular senescence still need to be addressed in details. In the present study, we established a replicative senescence model of HUVECs, and revealed metabolic changes of cells at different passages during cellular senescence. We observed continuously varying metabolic profiles, which were closely related to the increasing cell passages. Furthermore, we identified the significant metabolites primarily responsible for metabolic distinctions between the different groups of HUVEC cells, and quantitatively compared the aging-related changes of metabolite levels. In addition, we identified significantly altered metabolic pathways during cellular replicative senescence. To the best of our knowledge, this work represents the first metabonomic analysis for uncovering the global metabolic changes associated with cellular replicative senescence.

### A critical turning point during cellular replicative senescence

HUVEC cells at early passages (P2-P6) are extensively used as a classic in vitro model system for assessing physiological functions of vascular tissues. It is generally believed that HUVECs cultured more than P10 are no longer used due to the rapid loss of proliferation ability and obvious changes of morphology [21]. Consistent with this knowledge, our results indicate that a critical turning point occurs during the progression of replicative senescence of HUVECs. We found that the transformation occurred around P10. Relative to the younger P3 and P6 cells, the P10 cells displayed distinctly different cellular morphology and senescent status, and obviously lower proliferation capacity. Moreover, the metabolic properties of the P10 cells were significantly distinguished from those of the younger cells, as indicated by the greatly shifted metabolic profile, primarily changed metabolite levels and significantly altered metabolic pathways. In summary, a critical turning point existed around P10 during cellular senescence induced by continual passaging. This finding suggests that future studies of cellular aging intervention should take into account the fact that cellular aging is a gradually changing process. The critical turning point might act as an alternative time-point for starting the cellular aging intervention.

### Promoted oxidative stress during cellular replicative senescence

Numerous studies have shown that replicative senescence can be viewed as a result of a constant moderate stress such as stimuli from reactive oxygen species (ROS) to the cells [22]. Continuous cultivation of HUVEC cells could promote endogenous formation of ROS [23, 24]. In this study, we observed gradually decreased levels of several antioxidant molecules (glutathione, creatine and fumarate). This observation might reflect the weakened antioxidant defense system in senescent cells. Previous works have reported that glutathione and creatine could enhance the capacity of removing ROS, which play key roles in combating increased oxidative stress [25–27]. Additionally, fumarate could defense against cytotoxic effects of oxidative stress by activating the Nrf2 antioxidant response pathway [28]. We also observed that glycine and glutamate, which could act as building blocks of glutathione, were largely consumed during the progression of cellular senescence. These results suggest that cellular replicative senescence gradually results in the inability of the self-defense antioxidant system.

On the other hand, we observed that the levels of choline, PC and GPC were continually increased as HUVEC cells were serially passaged, which was in accordance with the fact that free radicals and ROS induced by cellular senescence could generate a lipid peroxidation process [29]. As components of phospholipids, these choline-containing compounds were crucial for structural integrity of cell membranes, and their increases could be ascribed to ROS-induced membrane damage [30]. In brief, the significant promotion of oxidative stress might account for the destabilization of lipid membranes during cellular senescence.

Despite the weakened antioxidant defense system, the cells exerted some replenishment to minimize the cellular damage. As one of the intracellular antioxidants, taurine was continuously increased from P3 to P10, and then was gradually decreased after P10. These data provided clear evidence that a taurine anaplerosis was involved in continuously promoted oxidative stress at the early stage of HUVECs senescence. Nevertheless, HUVEC cells were ultimately overwhelmed by the senescence-induced oxidative stress.

In line with the above-described results, the metabolic pathway analysis revealed that several metabolisms in P6, P10, P14 and P18 cells, including GSH metabolism, taurine and hypotaurine metabolism, and glycerophospholipid metabolism, were significantly altered relative to the young P3 cells. We also found that the significance of glycerophospholipid metabolism was gradually increased during cellular senescence, as indicated by the rising –ln(p) score from 15 to 30. These results show that cellular oxidative stress is promoted during replicative senescence. As reported by previous studies [14], cellular aging is accompanied by promoted oxidative stress. The metabolic changes of aging cells suggest that the molecular mechanisms of cellular replicative senescence are mostly associated with the decreases of some antioxidant metabolites, the aggravation of cell membrane instability and so on.

### Impaired energy metabolism during cellular replicative senescence

The redox balance maintained by antioxidant defense system is very important in metabolisms, especially energy metabolism as mitochondrial DNA stability is vulnerable to oxidative damages. In the present work, the process of HUVECs senescence showed significantly declining levels of energy metabolism-related metabolites, including AXP (adenine mono/di/tri phosphate), NAD^+^, and creatine. Indeed, there is growing evidence linking the change in cellular energy to the appearance of cellular senescence [15, 16]. Similar to our observation, Zwerschke et al. reported drastically decreased ATP levels in the processes of replicative senescence of human diploid fibroblasts (HDFs) and HUVECs [15, 16]. As well known, NAD^+^ and creatine play essential roles in ATP generation. NAD^+^ is a coenzyme in redox reactions by carrying electrons from one reaction to another [31], while creatine can facilitate recycling ADP to ATP via donation of phosphate groups [32]. Thus, the decreased levels of NAD^+^ and creatine limit the cellular energy flux, indicating a hampered oxidation respiratory in senescent HUVECs. Furthermore, NAD^+^ is necessary for the activity of sirtuins acting as NAD^+^ dependent deacetylases [33]. Previous studies have demonstrated that sirtuins are of great importance in regulating cellular aging through DNA damage repair, resistance to oxidative stress and so on [33, 34]. Consequently, the decreased NAD^+^ level might retard the senescence inhibition of sirtuins during cellular replicative senescence.

Besides those metabolites described above, some other metabolites in the tricarboxylic acid (TCA) cycle also showed significantly changed levels. Fumarate is an intermediate produced from the oxidation of succinate by the enzyme succinate dehydrogenase with malonate as a competitive inhibitor [35, 36]. We observed that fumarate was continually decreased and malonate was gradually increased during cellular senescence. These observations provide further confirmation that energy production is decreased during cellular replicative senescence.

As it is known, young cells are actively proliferating and generally exhibit high glycolytic rates to satisfy the energy demand compared with growth-arrested senescent cells [37]. More significantly, the lactate level was continually reduced during the procession of HUVECs senescence, despite the insignificantly changed glucose level. Taken all together, these results suggest that replicative senescence is characterized by a substantial loss of cellular energy currency owing to the reduction of both glycolysis and mitochondrial respiration.

Although senescent cells no longer achieve a large amount of energy for cell proliferation, a certain amount of energy is in demand for preserving normal cellular activities, including substance transportation, information convection, etc. To maintain energy homeostasis, consequently, large amounts of neutral amino acids (valine, isoleucine and glycine) were metabolized as alternative energy-generating sources during HUVECs senescence. Due to the declining metabolic rates during cellular aging, the levels of glutamine, aspartate and asparagine were gradually enhanced from P3 to P14. However, these metabolites were sharply decreased from P14 to P18. We speculate that the three metabolites might act as the last remaining sources for cellular energy, and finally enter into the TCA cycle through oxaloacetate and α-ketoglutarate.

Our results suggest that vascular aging might partially result from energy insufficiency in endothelial cells, which is associated with impaired energy metabolism and disturbed energy anaplerosis.

### Blocked protein synthesis during cellular senescence

Expectedly, both the promoted oxidative stress and impaired energy metabolism have several negative effects on HUVECs, in which protein synthesis block is the most prominent one. As previously reported, GSH participates in many metabolic and biochemical reactions such as DNA synthesis and repair, protein synthesis [38], amino acid transport [39], and enzyme activation [40]. The depletion of GSH caused by promoted oxidative stress could ultimately retard protein synthesis. The decreased ATP level could also hamper protein anabolism.

In addition to the metabolites related to anti-oxidation and energy supply, several amino acids (valine, isoleucine, glycine, proline, glutamate and pyroglutamate) were gradually decreased as HUVEC cells were sequentially passaged. These metabolites could be utilized as alternative sources of TCA cycle anaplerosis, providing the basic energy for cell survival. Low levels of these amino acids would hamper the anabolic state by stimulating proteolysis and inhibiting protein synthesis. Correspondingly, several significantly altered amino acid-related pathways were identified including glycine, serine and threonine metabolism; aminoacyl-tRNA biosynthesis; arginine and proline metabolism; glutamate and glutamine metabolism; alanine, aspartate and glutamate metabolism. These results indicate that disordered amino acids metabolisms might also significantly contribute to cellular replicative senescence, which could have adverse effects on protein synthesis.

In summary, the replicative senescence of HUVEC cells is a complicated physiological process closely associated with significantly impaired metabolisms. The NMR-based metabolomic analysis demonstrates that the replicative senescence of HUVEC cells is closely associated with cellular metabolic disorders, including promoted oxidative stress, impaired energy metabolism and blocked protein synthesis. Our results are beneficial to a deep understanding of metabolic changes during the progression of cellular replicative senescence, and may provide a new insight into the molecular mechanisms of vascular aging.

## MATERIALS AND METHODS

### Cell isolation and culture

Primary HUVEC cells, which were isolated from the umbilical cord of a neonate as described previously [41], were cultured in endothelial cell medium (ECM; ScienCell, USA), supplemented with 5% fetal bovine serum (FBS; ScienCell, USA), 1% endothelial cell growth supplement (ECGS; ScienCell, USA), 100 U/ml penicillin, and 100 U/ml streptomycin. The cells were incubated at 37 °C with 5% CO_2_ in a humidified atmosphere. The experimental protocol for HUVECs isolation was approved by the ethics committee of the Affiliated ZhongShan Hospital of Xiamen University, China. The enrolled parturient was negative for human immunodeficiency virus and hepatitis B virus, and signed the written informed consent for providing her umbilical cord.

At passage 2 (P2), cells were seeded into 10-cm culture dishes at the density of 5 × 10^5^ cells/dish. After reaching 80% confluence, cells were harvested and reseeded into new culture dishes with the same starting number and cultured repeatedly till passage 18 (P18). Cells were passaged every 2-3 days at early passages, and 5-6 days for later passages. Morphological changes were observed with an inverted phase-contrast microscope. The number of population doublings (PD) was calculated at each time point of passaging with the following equation: PD = (lg F - lg I)/0.301, where F and I were the final counted cell numbers and the initial counted cell numbers, respectively. The population doublings level (PDL) was the accumulated PD at the end of each subculture, which was calculated using the following formula: PDL_(end)_ = PDL_(initial)_ + PD, where PDL = 0 for P2 cells. Cells of passages 3, 6, 10, 14 and 18 (P3, P6, P10, P14 and P18) were thawed and recovered for all experiments.

### Senescence-associated β-galactosidase (SA-β-gal) staining

The senescent status of HUVEC cells was verified by a senescence detection kit (BioVision, USA) according to the manufacturer’s instruction. Briefly, cells grown on 12-well culture plates were washed once with PBS (Hyclone, USA) after medium removal, and then fixed with the fixative solution for 12 min at room temperature. After being washed twice with PBS, the cells were incubated in freshly prepared staining solution for 12 h at 37 °C with the plate covered. The rate of positively stained cells (blue cells) to total cells was calculated in 10 randomly chosen microscopic fields under ×100 objective magnification [42].

### Real-time growth profile

HUVEC cells were seeded in a E-Plate 16 Device (ACEA Biosciences, USA), and the growth kinetics of the cells was monitored continuously using a RTCA S16 instrument (ACEA Biosciences, USA) at 37 °C in a humidified atmosphere with 5% CO_2_. The RTCA S16 system utilized E-plate containing interdigitated microelectrodes on the bottom of the plate, to detect local ionic changes during cell proliferation. The proliferation rate was measured as electrode impedance. Cell sensor impedance was expressed as an arbitrary unit termed cell index (CI), which was defined as (Z_i_ – Z_0_)/15, where Z_i_ was the impedance at a given point of time in the experiment, and Z_0_ was the impedance at the beginning of the experiment.

### Immunofluorescence assay

HUVEC cells were prepared in Millicell EZ SLIDE 8-well plates (Millipore, USA). Cells were fixed with 4% formaldehyde at 4 °C for 30 min, washed thrice in PBS, and permeabilized with 0.3% Triton X-100 for 10 min. Then, the cells were blocked with 5% BSA solution for 60 min at room temperature, incubated with the primary antibody for 3 h, and with the secondary antibody for 1.5 h at 37 °C. Cell nuclei were counterstained with 4’, 6-diamidino-2-phenylindole (DAPI; Invitrogen, USA). Cells were imaged and analyzed with a ZEISS LSM 780 Confocal Laser Scanning Microscope (Carl Zeiss, Germany). The following three antibodies were used for biochemical characterizations of the cells: anti-F8 rabbit polyclonal (Sangon Biotech, China; 1:100 dilution), anti-CD31 rabbit polyclonal (Sangon Biotech, China; 1:100 dilution), and anti-vWF rabbit polyclonal (Abcam, USA; 1:150 dilution). FITC-conjugated mouse anti-rabbit IgG was used as the secondary antibody for detecting F8 and CD31, and donkey anti-rabbit IgG H&L (Alexa Fluor® 647) was used for detecting vWF.

### Intracellular metabolites extraction

To harvest a similar number of HUVEC cells in different group for the metabolomics experiments, cells of P3, P6, P10, P14 and P18 were seeded with gradually changed densities as 3 × 10^5^, 4 × 10^5^, 5 × 10^5^, 7 × 10^5^, 1 × 10^6^ cells/dish, respectively. After 5 days of culture, approximately 2×10^6^ cells/dish were harvested in each group. Cells were quenched by a direct cell quenching method as described previously [43]. Briefly, cells were quickly washed thrice with ice-cold PBS (pH 7.4) after culture medium removal. The residual PBS was entirely exhausted by vacuum suction. Then, 3 ml of cold methanol was immediately added into the culture dish, and the cells were scraped, collected and transferred into a 15-ml centrifuge tube. For extraction, both 3 ml of HPLC grade chloroform and 2.5 ml of ultrapure H_2_O were also added and mixed, according to a dual phase extraction procedure described by Viant et al [44]. After 30 min of laying aside, the extract solutions were centrifuged at 12000 g for 15 min at 4 °C to separate two phase extracts. Finally, the aqueous phase was condensed with a Nitrogen Blowing Concentrator and lyophilized by a vacuum freezing dryer.

### Sample preparation and NMR measurements

Lyophilized powder of each aqueous extract sample was then dissolved in 550 μl of NMR buffer containing 50 mM K_2_HPO_4_/NaH_2_PO_4_ (pH 7.4), 0.01mM sodium 3-(trimethylsilyl)-propionate-2,2,3,3-d4 (TSP), 10% D_2_O and 0.02% NaN_3_. D_2_O was used for the field- frequency lock, and TSP was used to provide the chemical shift reference (δ 0.00). Then, the samples were vortexed, centrifuged at 12000 g for 15 min at 4 °C to remove any insoluble components. At last, the supernatants were transferred into 5-mm NMR tubes for further analysis. All ^1^H NMR spectra were recorded on a Bruker Avance III 850 MHz spectrometer (Bruker BioSpin, Germany) at 25 °C using the pulse sequence NOESYPR1D [(RD)-90°-t_1_-90°-τ_m_-90°-Acq] with water suppression. The following experimental parameters were used: spectral sweep width, 20 ppm; total relaxation recovery delay (RD), 4 s; a short delay t_1_, 4 μs; the mixing time (τ_m_), 10 ms; acquisition time per scan (Acq), 1.88 s; data points, 64 K; number of the scans, 128.

### NMR data preprocessing

All free induction decay signals (FIDs) were processed using the Topspin-2.1 software (Bruker Biospin, Germany) with a standard Fourier transformation (FT) procedure following manual phase and baseline correction. The FIDs were multiplied with an exponential line-broadening factor of 0.3 Hz. Chemical shifts were referenced to the methyl-group of TSP at 0 ppm. Alignments of NMR spectral lines were performed by using the MestReNova software (Version 9.0, Mestrelab Research S.L., ESpain). After removing the regions of δ 4.59–5.20 and δ 3.37–3.38 ppm to eliminate the influences of water and methanol, the spectra were segmented at δ 0.003 intervals across the chemical shif range of 0.80–9.40 ppm. To reduce concentration differences among the samples, NMR spectral integrals of metabolites were normalized to the total spectral integrals by using MatLab (Version 2011b; Math Works, USA). Metabolites were identified by a combination of the Chenomx NMR Suite (Version 8.0, Chenomx Inc., Canada), the Human Metabolome Data Base (HMDB, http://www.hmdb.ca/), and relevant published references [45], confirmed by 2D NMR spectra including ^1^H-^13^C HSQC and ^1^H-^1^H TOCSY.

### Multivariate pattern recognition analysis

For multivariate data analysis, the normalized spectral data were imported into the SIMCA-P software (Version 12.0, Umetrics AB, Umeå, Sweden), and scaled by Pareto scaling to increase the importance of low-level metabolites without significant amplification of noise. The unsupervised principal component analysis (PCA) was performed to observe the grouping trends, highlight outliers, and show clusters among the groups. Moreover, hierarchical cluster analysis with Pearson distance measure and Ward clustering algorithm was conducted on the normalized spectral data to further confirm the metabolic clusters using the module of Statistical Analysis provided by the MetaboAnalyst 4.0 webserver (http://www.metaboanalyst.ca) [46]. In hierarchical cluster analysis, each sample worked as a separate cluster initially and the algorithm proceeded to combine them until all samples belonged to one cluster.

Furthermore, the supervised orthogonal projection on latent structure with discriminant analysis (OPLS-DA) was utilized to improve the classification of the different groups and screen potential variables significantly responsible for the metabolic distinctions between different groups. The S-plot of the OPLS-DA model was used to identify significant metabolites primarily contributing to the metabolic separation between different groups based on three criteria: the absolute value of w[1] > 0.06, the absolute value of p(corr)[1] > 0.75, and VIP ≥ 1.00.

### Metabolite quantitative analysis

We used relative integrals of the significant metabolites identified from the OPLA-DA models to quantitatively analyze metabolite levels in the five groups of HUVEC cells (n = 8 for each group). For metabolites related to highly overlapping peaks, non-overlapping peaks were selected to accurately calculate spectral integrals. Averaged metabolite integrals were expressed as Mean ± Std. Error for each group. Variations were statistically calculated by utilizing one-way analysis of variance (ANOVA) followed by Tukey’s multiple comparison test with the Bonferroni correction using the SPSS software (Version 19.0, Chicago, IL, USA). A total of 10 independent hypotheses were tested for multiple comparisons among the five groups of HUVECs. The statistical significance level used for each hypothesis separately is 1/10 times what it would be if only one hypothesis were tested. Thus, we identified differential metabolites with statistical significances of *p* < 0.005.

### Metabolic pathway analysis

We conducted metabolic pathway analysis to identify significantly altered metabolic pathways during the progression of cellular replicative senescence base on the significant metabolites identified from the OPLS-DA analysis, using the module of Pathway Analysis provided by the MetaboAnalyst 4.0 webserver. The following parameters were selected for the pathway topological analysis, including the ‘Homo sapiens (human)’ library, the default ‘Global Test’ and ‘Relative Betweenness Centrality’. Metabolic pathways with -ln(*p*) scores > 8 and pathway impact values > 0.03 were identified to be significantly altered metabolic pathways.

## Supplementary Material

Supplementary Figures

Supplementary Tables
