# Supplementary material for: NMR-based metabonomic analysis of HUVEC cells during replicative senescence
Source: Aging (Albany NY). 2020 Feb 17;12(4):3626–46. doi: 10.18632/aging.102834 (PMC7066908; doi:10.18632/aging.102834)
Supplement: Supplementary Figures [file aging-12-102834-s002..pdf]

SUPPLEMENTARY FIGURES

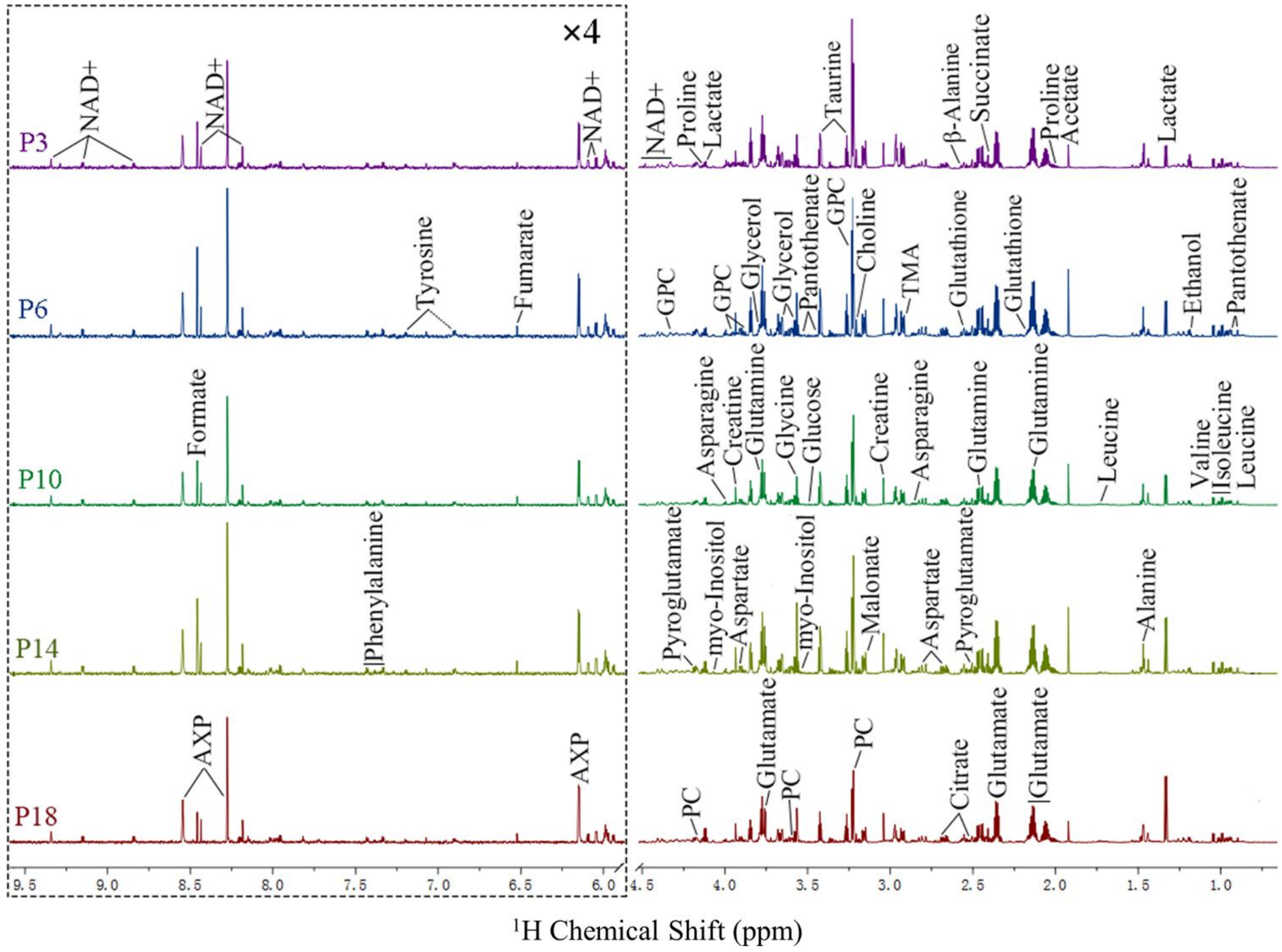

Supplementary Figure 1. Typical 1D ^{1}H NMR spectra recorded on aqueous extracts of HUVEC cells at different passages. Identical vertical scales were applied in all the NMR spectra recorded on a Bruker Avance 850 MHz spectrometer. The water region (4.5-5.6 ppm) was removed. The region of 5.6-9.5 ppm (in the dashed box) was magnified 4 times compared with the corresponding region of 0.5-4.5 ppm for the purpose of clarity. Abbreviations: PC, O-Phosphocholine; GPC, sn-Glycero-3-phosphocholine; AXP, adenine mono/di/tri phosphate; NAD$^+$, nicotinamide adenine dinucleotide.

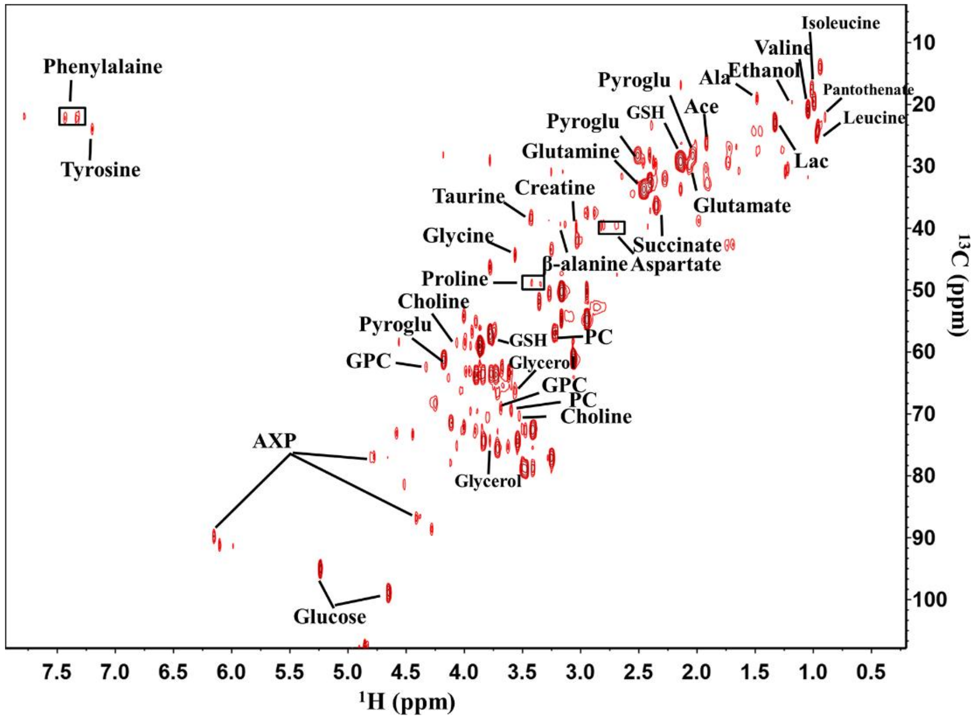

Supplementary Figure 2. Typical 2D ^{1}H-^{13}C HSQC spectrum of aqueous extracts derived from HUVEC cells. The spectrum was recorded at 25 °C on a Bruker Advance III 850 MHz NMR spectrometer.

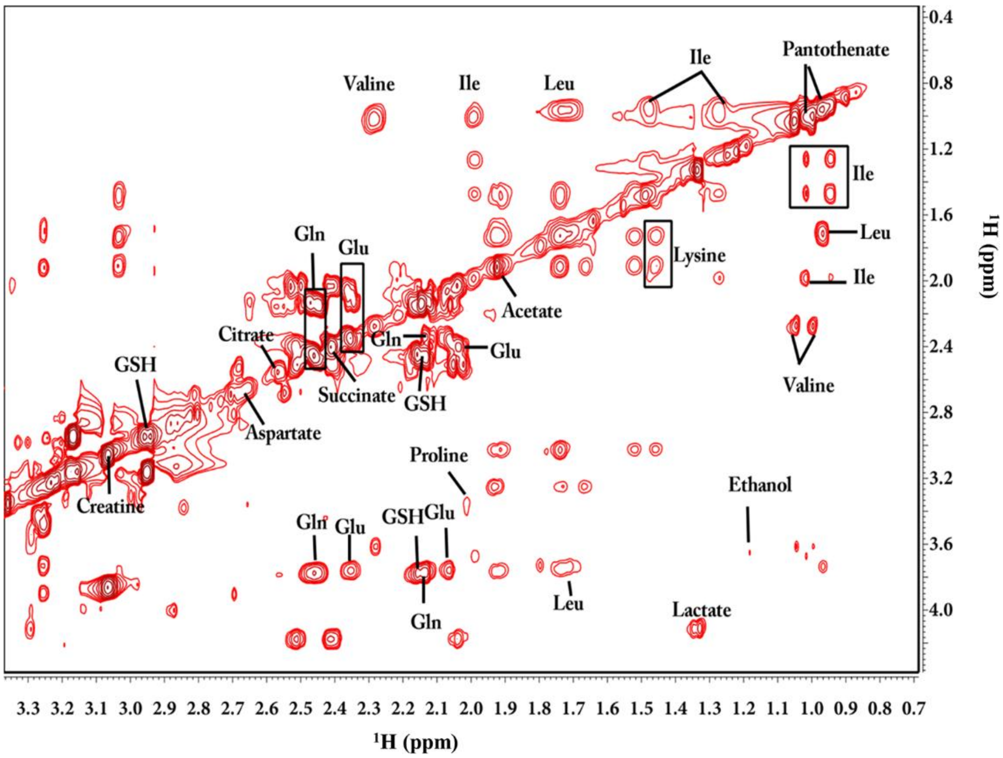

Supplementary Figure 3. Region (0.7-3.3 ppm) of a typical 2D ^{1}H-^{1}H TOCSY spectrum of aqueous extracts derived from HUVEC cells. The spectrum was recorded at 25 °C on a Bruker Advance III 850 MHz NMR spectrometer.

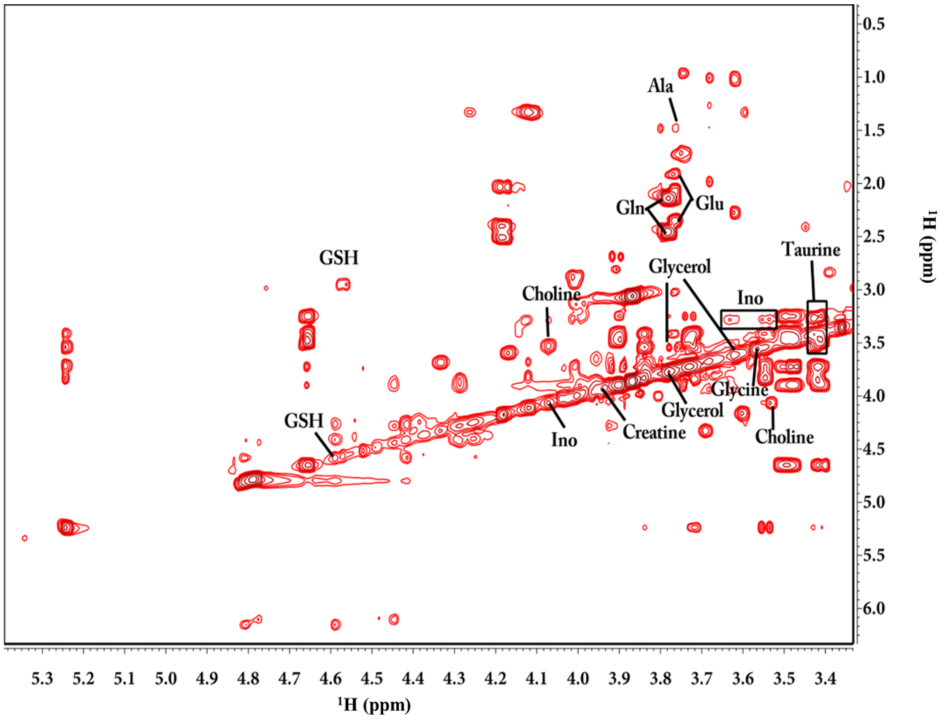

Supplementary Figure 4. Region (3.4-5.3 ppm) of a typical 2D ^{1}H-^{1}H TOCSY spectrum of aqueous extracts derived from HUVEC cells. The spectrum was recorded at 25 °C on a Bruker Advance III 850 MHz NMR spectrometer.

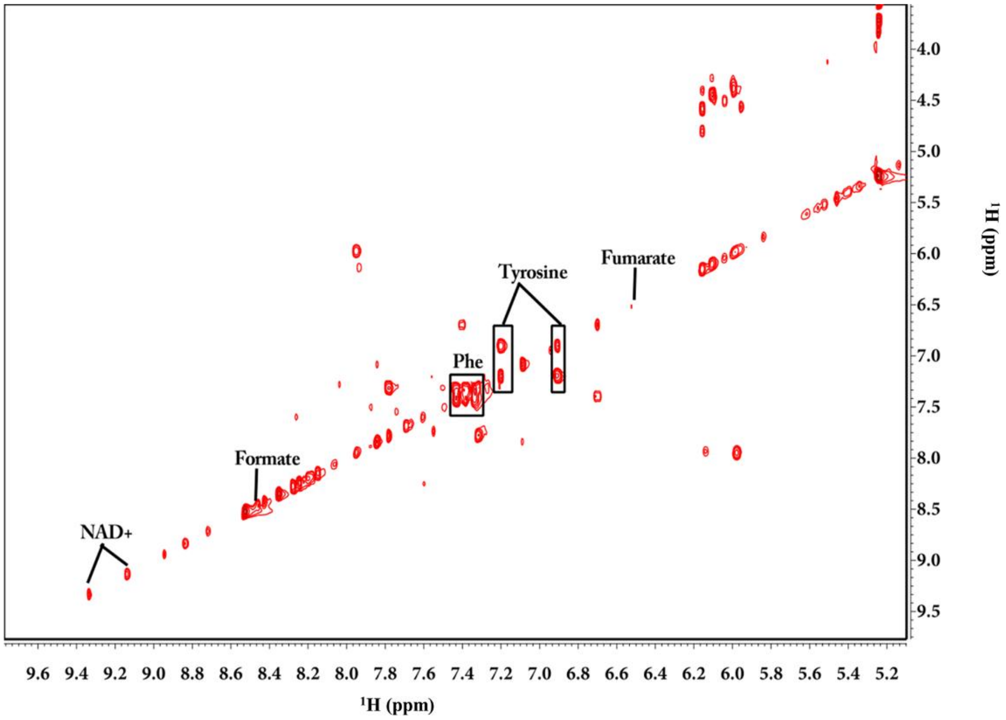

Supplementary Figure 5. Region (5.2-9.6) ppm of a typical 2D ^{1}H-^{1}H TOCSY spectrum of aqueous extracts derived from HUVEC cells. The spectrum was recorded at 25 °C on a Bruker Advance III 850 MHz NMR spectrometer.
