# Supplementary material for: NMR-based metabonomic analysis of HUVEC cells during replicative senescence
Source: Aging (Albany NY). 2020 Feb 17;12(4):3626–46. doi: 10.18632/aging.102834 (PMC7066908; doi:10.18632/aging.102834)
Supplement: Supplementary Tables [file aging-12-102834-s001..pdf]

SUPPLEMENTARY TABLES

Supplementary Table 1. Parameters for assessing the qualities of OPLS-DA models of the four groups of HUVEC cells vs. the P3 group.

	OPLS-DA model			
	R^2X	R^2Y	Q^2	CV-ANOVA
P6 vs. P3	0.279	0.704	0.500	0.011
P10 vs. P3	0.577	0.995	0.935	7.33e-5
P14 vs. P3	0.564	0.996	0.977	5.66e-9
P18 vs. P3	0.579	0.995	0.989	2.04e-13

Supplementary Table 2. Characteristic metabolites identified from the OPLS-DA models of the four groups of HUVEC cells vs. the P3 group.

Metabolite	P6 vs. P3		P10 vs. P3		P14 vs. P3		P18 vs. P3	
	VIP[a]	Change[b]	VIP	Change	VIP	Change	VIP	Change
Isoleucine	-	-	1.077	↓	1.051	↓	1.110	↓
Valine	1.536	↓	1.321	↓	1.198	↓	1.155	↓
Lactate	1.718	↓	1.405	↓	1.246	↓	1.276	↓
Proline	-	-	1.319	↓	1.213	↓	1.286	↓
Glutathione	-	-	-	-	1.059	↓	1.268	↓
Glutamate	-	-	-	-	1.139	↓	1.275	↓
Glutamine	-	-	1.009	↑	1.188	↑	-	-
Aspartate	-	-	1.269	↑	1.061	↑	-	-
Asparagine	-	-	-	-	1.238	↑	-	-
Creatine	-	-	-	-	1.255	↓	1.297	↓
Malonate	-	-	1.032	↑	1.094	↑	1.236	↑
β-Alanine	-	-	1.372	↓	1.262	↓	1.233	↓
Choline	-	-	-	-	1.240	↑	1.289	↑
PC	1.982	↑	1.500	↑	1.277	↑	1.268	↑
GPC	-	-	1.243	↑	1.267	↑	1.305	↑
myo-Inositol	1.774	↓	1.293	↓	1.212	↓	1.259	↓
Glycine	1.713	↓	1.398	↓	1.149	↓	1.255	↓
Fumarate	-	-	-	-	1.164	↓	1.286	↓
AXP	-	-	1.218	↓	1.090	↓	1.154	↓
NAD^+	-	-	-	-	1.245	↓	1.238	↓
Pantothenate	1.798	↓	-	-	-	-	-	-
Taurine	1.633	↑	1.259	↑	-	-	-	-
Phenylalanine	-	-	1.061	↓	-	-	-	-
Pyroglutamate	-	-	-	-	-	-	1.075	↓

[a] VIP: Variable importance in the projection.
[b] "↑/↓" mean significantly increased/decreased metabolite concentrations relative to the P3 group with statistical significance $p < 0.05$.
